# What Role Do Inflammatory Cytokines Play in Cancer Cachexia?

**DOI:** 10.7759/cureus.26798

**Published:** 2022-07-12

**Authors:** Jyothirmai Malla, Anam Zahra, Sathish Venugopal, Tharun Yadhav Selvamani, Shoukrie I Shoukrie, Ramaneshwar Selvaraj, Ravneet K Dhanoa, Ranim K Hamouda, Jihan Mostafa

**Affiliations:** 1 Internal Medicine, California Institute of Behavioral Neurosciences & Psychology, Fairfield, USA; 2 Surgery, California Institute of Behavioral Neurosciences & Psychology, Fairfield, USA; 3 Neurology, California Institute of Behavioral Neurosciences & Psychology, Fairfield, USA; 4 General Surgery, California Institute of Behavioral Neurosciences & Psychology, Fairfield, USA; 5 Orthopaedics and Traumatology, California Institute of Behavioral Neurosciences & Psychology, Fairfield, USA; 6 Internal Medicine/Family Medicine/General Surgery, California Institute of Behavioral Neurosciences & Psychology, Fairfield, USA

**Keywords:** adipose tissue, skeletal muscle, cytokines, anorexia, cachexia

## Abstract

A tumor extends its effects beyond its local site, and one such effect is cancer cachexia which is caused by a state of systemic inflammation in response to cancer. Though the prominent effect of cancer cachexia is seen on skeletal muscles, it shows deterioration in other organs’ smooth muscle, adipose tissue, blood, bone marrow, liver, and immunity. Interleukin (IL)-6 plays an imminent role along with tissue necrosis factor-alpha, IL-1 beta, interferon-gamma, myostatin, adiponectin, growth differentiation factor-15, activin A, etc. These cytokines through nuclear factor-kappa beta, mitogen-activated protein kinase, suppressor of mothers against decapentaplegic, and Janus activated kinase/signal transducer and activator of transcription pathway activate genes inducing ubiquitin-proteosome system and reactive oxidative species. Apart from these, they participate in causing anemia and immunosuppression. Adipose tissue acts as a source of cytokines and place of action of cytokines leading to lipolysis. Moreover, these cytokines act at the hypothalamic-pituitary-adrenal axis change metabolism and add to anorexia which already exists in cancer patients. The involvement of multiple cytokines necessitates the development and testing of anti-cytokines in combinations.

## Introduction and background

According to the World Health Organization (WHO), most cancers account for approximately 10 million deaths withinside the year or one in six deaths worldwide. Breast cancer is the leading cancer type but the cancer that is causing the highest number of deaths is lung cancer [1]. Many factors such as genetics, senescence, lifestyle, environment, and infections are responsible for this burden of cancer on the world [2].

Cancer cachexia is a complex multifactorial syndrome that affects about half of cancer patients and three-fourths of those with advanced cancer [3-5]. They are also responsible for one-fourth of the deaths in cancer patients [6-8]. Additionally, it differs from the form of most cancers. It is determined to be extra profound in lung cancer, colorectal cancer, and melanoma. It is characterized by severe weight loss, extensive skeletal and visceral muscle loss, adipose tissue loss, and negative energy balance. It is an interplay of tumor growth, inflammatory cytokines, neuropeptides, inflammatory proteins, hormones, and anorexia [6,9].

According to international consensus, cancer cachexia is defined as “weight loss of more than 5% over past six months in absence of starvation and/or BMI less than 20 kg/m² and more than 2% ongoing weight loss and/or sarcopenia and more than 2% ongoing weight loss” [10]. It is critical to distinguish between starvation and cachexia. The major difference is that starvation is fat loss and can be reversed by nutritional interventions. This does not apply to cachexia with more muscle loss than fat loss and cannot be reversed with nutritional interventions [11].

Cancer cachexia is usually diagnosed only after a weight loss of 7-15% [3] and decreases the quality of a patient’s life. It is screened considering various factors such as weight loss in the last six months, upper arm circumference, triceps skinfold thickness, strength in the handgrip, C-reactive protein (CRP) levels, and screening tools such as malnutrition universal screening tool (MUST) [12]. To better understand and treat cancer cachexia, we performed a review of research literature in the last 20 years on the role of inflammatory cytokines in cancer cachexia.

## Review

Source of cytokines

**Figure 1:**
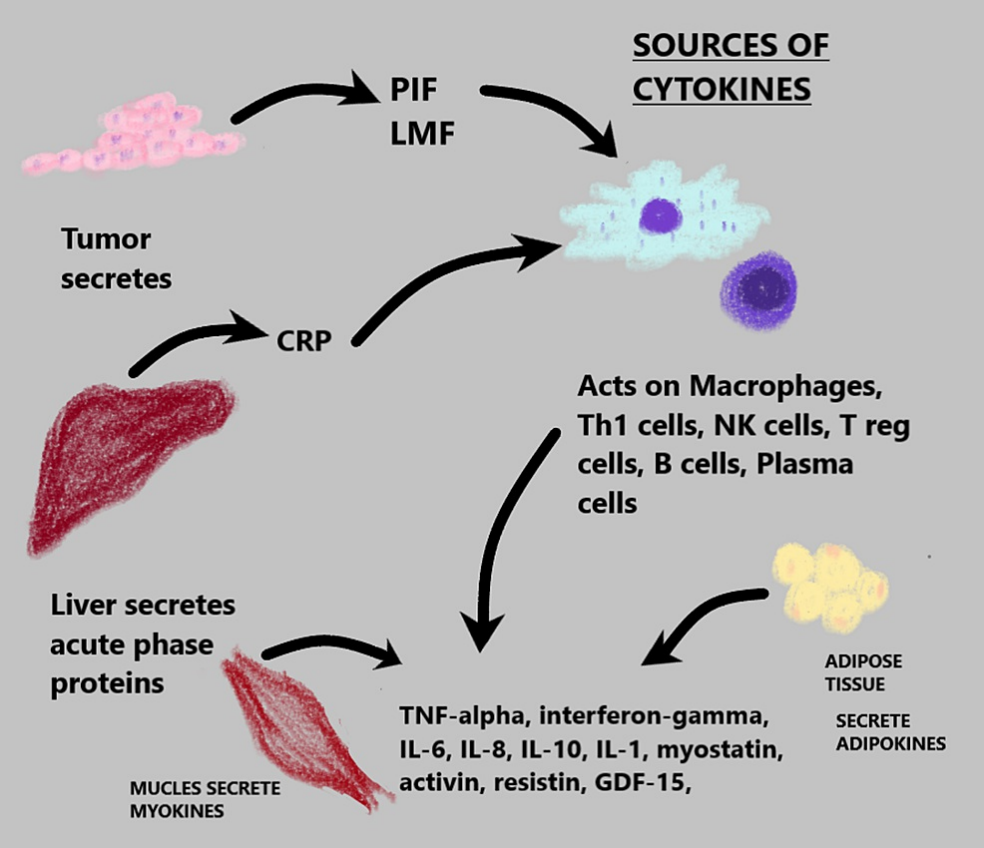
The various sources of inflammatory cytokines which result in cancer cachexia. Source: Image created by the authors. CRP: C-reactive protein; GDF-15: growth and differentiation factor-15; IL: interleukin; LMF: lipid mobilizing factor; NK cells: natural killer cells; PIF: proteolysis-inducing factor; Th1 cells: T-helper 1 cells; TNF-alpha: tissue necrosis factor-alpha; T reg cells: regulatory T cells

Cancer cachexia is a state of systemic inflammation, and inflammatory cytokines play the first-rate component in this. Tumors express proteolysis-inducing factor (PIF) that activates the nuclear factor-kappa B (NF-κB) pathway which leads to the activation of targeted genes to produce cytokines and chemokines resulting in inflammation. It activates the signal transducer and transcription factor 3 (STAT 3) pathway and leads to the synthesis and release of interleukin (IL)-8 (IL-8), IL-6, tissue necrosis factor-alpha (TNF-α), interleukin-1-beta (IL-1beta), monocyte chemoattractant protein-1 (MCP-1), and interferon-gamma (IFN‐γ) [13]. As a response to tumor growth immune cells such as the cluster of differentiation 8 T cells (CD8 T cells), T-helper 1 cells (Th1 cells), natural killer cells (NK cells), macrophages M1, M2, T regulatory cells (T reg cells), myeloid-derived suppressor cells, B cells, plasma cells, mast cells, neutrophils, and eosinophils. Each of these cells is responsible for secreting diverse kinds of cytokines; for example, M1 secretes IL-6, IL-12, TNF-α, and IL-23; M2 secretes IL-10 [9]. In addition to these, white adipose tissue (WAT) is an excellent source of adipokines such as adiponectin, leptin, TNF-α, IL-6, IL-8, IL-10, vascular endothelial growth factor (VEGF), MCP-1, and myocytes also produce myokines such as myostatin, growth and differentiation factor 15 (GDF-15), and activin-A [14-16]. Figure 1 depicts the different possible sources of inflammatory cytokines in cancer cachexia.

Effect of cytokines on muscle loss

Muscle loss is the most imperative feature of cachexia. It not only affects skeletal muscle but also visceral muscle. In addition to losing weight and strength for day-to-day activities, it also leads to cardiac and diaphragmatic muscle damage leading to death due to cardiac failure and respiratory arrest, respectively [17]. Table 1 lists the different methods by which pro-inflammatory cytokines cause muscle loss.

**Table 1:** Summary of various ways by which inflammatory cytokines cause muscle loss. C-EBPbeta: CCAAT/enhancing binding protein beta; HPA: hypothalamic-pituitary-adrenal axis

Pro-inflammatory cytokines affect muscle loss by
Stimulating HPA to increase cortisol
Increasing synthesis of 11-beta-HSD which activate inactive glucocorticoids
Activating ubiquitin-proteasome system for proteolysis
Increasing reactive oxygen species
Activating caspase-3 for myocyte apoptosis
Activating cathepsins in the autophagy-lysosomal pathway
Inhibiting myogenesis and proliferation by C/EBPbeta
Inhibiting early myoblast differentiation by reducing Myo-D mRNA
Inhibiting mitochondrial biogenesis
Reducing muscle creatine kinase
Bringing in redox imbalance
Inducing accumulation of ceramide in myocyte
Increasing atrophy vulnerable fast fibers

There are a few ways by which cytokines cause muscle loss. One of these is by augmenting the synthesis of cortisol. Cytokines such as TNF-α, IL-1-beta, and IL-6 act on the hypothalamic-pituitary-adrenal axis (HPA) to release corticotropin-releasing hormone (CRH) from the paraventricular nucleus. CRH acts on the anterior pituitary to release adrenocorticotropic hormone (ACTH) that binds to mineralocorticoid 2 (MC2) receptors on the adrenal cortex to initiate adrenal cortisol synthesis. 11-beta-hydroxysteroid dehydrogenase-1 (11-beta-HSD-1) is synthesized in the liver, adipose tissue, muscle, bones, and immune cells. 11-beta-HSD-1 mediates the conversion of inactive glucocorticoids into active cortisol. It has been proven that cortisol causes muscle wasting [11].

Cytokines contribute to muscle loss by increasing muscle proteolysis and diminishing muscle synthesis. Muscle proteolysis produces energy substrates for the growing needs of tumor growth, such as glutamine is used in the synthesis of nucleic acids which is essential for rapidly generating tumor cells. Similarly, alanine derived from this process participates in liver gluconeogenesis for the production of glucose which becomes the energy source for tumor cells [18]. IL-6 is a soluble pleiotropic IL involved in both anabolic and catabolic pathways. Over 100 ng/mL physiological level is required to participate in catabolic ways [3]. Along with IL-6, TNF-α, also named cachectin, IL-1-beta, and IFN‐γ activate the NF-κB pathway, promote the transcription of ubiquitin-proteasome E3 ligase [19,20], and responsible for muscle proteolysis

NF-κB is upregulated rapidly in the nucleus [4]. It is increased in the nucleus via the pathway of the interaction of cytokines with surface proteins which recruit a family of intracellular adaptor proteins [11]. It decays rapidly and migrates back to the cytoplasm after inducing transcription factors responsible for the ubiquitin-proteasome system (UPS) by using activation of gene muscle ring finger-1 (MuRF-1) and muscle atrophy F box (MAFbx)/Atrogin-1 gene.

The ubiquitin-proteasome system is proteasome-mediated proteolysis where protein is marked with a ubiquitin chain [19]. It includes three essential enzymes. E1 enzyme that is required for ubiquitin activation, E2 for conjugation, and E3 act as ligating enzymes [21]. E3 ligase genes are MuRF-1 and MAFbx/Atrogin-1 [22]. This enzyme complex binds to proteins and converts them into polyubiquitin protein which is transferred by utilizing ATP to 26s proteasome complex for degradation [11]. Although E3 ligase exists in two types, namely, E3a-I and E3a-II, E3a-II is more involved in muscle proteolysis and is upregulated by the effect of cytokines [21].

MuRF-1 and Atrogin-1 gene activation with UPS muscle proteolysis can also occur via Janus kinase (JAK)/signal transducer and activator of transcription (STAT) and mitogen-activated protein kinase (MAPK) signally [19]. IL-6 interacts with IL6R/GP130 on the cell surface to recruit a series of intracellular receptors to activate JAK tyrosine kinase. JAK, a receptor-bound protein, undergoes conformational changes to activate the STAT by phosphorylation. STAT carries the signal to the nucleus and permits DNA transcription. This JAK/STAT signal is also followed by other cytokines such as IFN‐γ and leucocyte inhibiting factor (LIF). Another signaling pathway leading to UPS proteolysis by activating MuRF-1, and Atrogin-1 is the mitogen-activated protein kinase/extracellular signal-regulated kinase (MAPK/ERK) pathway by different cytokines such as IL-1, TNF-α, IL-6, and cellular stress [11].

In addition, muscle damage also occurs by autophagy, myotube atrophy, and myocyte apoptosis [19]. Autophagy is the removal of damaged, misfolded, and senile proteins and organelles by lysosomes [23]. Cytokines acting through the JAK/STAT pathway increase myostatin/growth and differentiation factor-8 (GDF-8) which is a member of the transforming growth factor-beta (TGF-beta) family [11]. TGF-beta family cytokines such as activin, growth and differentiation factor 11 (GDF-11), and myostatin bind to activin receptors such as kinase (ALK)-5, ALK-6, and activin receptor 2B (ACVR2B) to activate the small mothers against decapentaplegic (SMAD) pathway by phosphorylation of SMAD2/3 [19]. This results in the activation of caspase-3 and Forkhead Box (FOXO)3 gene [11,19,23]. Caspases-3 are responsible for myocyte apoptosis by proteolytic activity on actin, myosin, and the myofibrillar protein [19,20]. FOXO3 gene regulates the expression of gene ATG7 (autophagy-related) and the expression of cathepsins B and L which are essential for the lysosomal degradation of myocyte proteins [19,24]. Cytokines acting through the NFkB pathways like IL-6, IL-1, TNF-α, and IFN‐γ also activate the FOXO3 gene activating the autophagy-lysosome pathway which breaks down proteins into amino acids.

However, inflammatory cytokines cause muscle loss by inhibiting myocyte regeneration, proliferation, and differentiation. The last step in muscle regeneration requires satellite cells in which few remain in the quiescent stage while the remaining multiply and differentiate into myocytes. Early differentiation requires MyoD protein and late differentiation needs MyoG [11,25]. FOXO produced via NF-kB and SMAD pathways reduces the MyoD mRNA expression pausing the early differentiation of myoblasts [11]. Besides this TNF-α and IFN‐γ induce iNOS (inducible nitric oxide synthase) which converts L-arginine to citrulline producing nitrogen monoxide (NO) which reacts with oxygen to produce ONOO^-^. This induces oxidative stress, muscle fiber loss, and a reduction in mRNA levels of MyoD [5,24]. MAPK pathway additionally inhibits the proliferation and differentiation of myocytes. CCAAT/enhancer-binding proteins-6 beta (C/EBPb) is a major regulator of myoblast and is activated by IL-6 and IL-1, resulting in myogenesis inhibition and muscle fiber size reduction [8,26].

IL-6, TNF-α, and TGF-beta also reduce mitochondrial biogenesis, which reduces mitochondrial function and ATP synthesis in myocytes [23]. On the other hand, it is produced by TNF-α by inducing inducible nitric oxide synthase (iNOS) to inhibit the JUN-D gene, thereby declining the muscle creatinine kinase which is essential for creating ATP from phosphocreatine and preserving ATP reserves. These pave the way for loss of muscle power and function [24]. GDF-15 is another cytokine belonging to the TGF-beta family which is increased in cancer cachexia and is responsible for the regulation of lean body mass and body weight [15]. TNF-α also decreases the ceramide kinase reducing the conversion of ceramide to ceramide-1-phosphate leading to ceramide accumulation and resulting in myotube atrophy [26]. In addition to these effects, IL-6 brings about changes in redox balance in muscle tissue by inhibiting the anti-oxidant work and increasing the fast fibers in muscle which are more prone to atrophy [27].

Effect of cytokines on adipose tissue loss

Adipose tissue is involved in both metabolism and immunity. WAT is a major source of adipokines such as adiponectin, leptin, VEGF, MCF-1, TNF-α, IL-6, and IL-8 [14]. WAT is also involved in the synthesis of hormones, autocoids, and fatty acids [2]. In cancer cachexia, WAT is lost and replaced by brown adipose tissue which is characterized by rich mitochondria leading to negative energy balance [20]. Subcutaneous adipose tissue is primarily affected. This adipose tissue loss is due to anorexia and the action of inflammatory factors. IL-6, TNF-α [23], IFN‐γ, and IL-1-beta activate hormone-sensitive lipase (HSL) and adipose triglyceride lipase (ATGL) resulting in free fatty acids (FFA). These FFA try to meet the energy requirements of the body lost due to resting energy expenditure. In addition, FFA activate macrophages aggregating the inflammation. Saturated fatty acids also behave as ligands similar to toll-like receptors (TLR4) to increase pro-inflammatory cytokines [28]. Tumor cells secrete another cytokine, namely, lipid mobilizing factor (LMF) which is homologous to zinc-alpha-2-glycoprotein (Zn-alpha-2-glycoprotein). Zn-alpha-2-glycoprotein is involved in multiple functions and was discovered to stimulate adenylate activity to increase lipolysis [6]. Table 2 provides a summary of different ways by which cytokines result in a reduction in adipose tissue.

**Table 2:** Summary of various ways by which cytokines result in adipose tissue loss. ATGL: adipose triglyceride lipase; HSL: hormone-sensitive lipase

Pro-inflammatory cytokines get involved in adipose tissue loss by
Converting white adipose tissue into brown adipose tissue
Activating HSL and ATGL
Mimicking zinc-alpha2-glycoprotein

Effect of cytokines on fibrosis

Cancer cachexia is a chronic state of systemic inflammation that leads to fibrosis of the extracellular matrix. TGF-beta initiates the SMAD pathway by phosphorylation of SMAD2/3 target genes causing the differentiation of fibroblasts into myofibroblasts. Cytokines such as TNF-α, IL-1, IL-13, and TGF-beta are responsible for stimulating the multiplication and endurance of these cells. As subcutaneous adipose tissue and adipocyte size reduce, fibrotic areas are formed. There is growth in collagen fiber, elastic fiber, and fibronectin. Type I and III collagen multiply with fibrosis [7].

Effect of cytokines on anorexia

Anorexia refers to the loss of appetite associated with nutritional deficit [11,29]. Several factors such as dysphagia, nausea, vomiting, gastrointestinal obstruction, decrease in bowel movements, side effects of medications such as opioids used for pain relief, chemo and radiotherapy, depression, and anxiety contribute to anorexia in cancer patients [6]. The energy needs of a patient increase with tumor growth but anorexia leads to further energy deficit resulting in the lipolysis of adipose tissue and proteolysis of muscle.

The paraventricular nucleus (PVN) and arcuate nucleus express receptors for cytokines such as IL-1beta, IL-6, TNF-α, and LIF [11]. These cytokines mimic leptin negative feedback on the hypothalamus and exert a cachexic effect [6]. The hypothalamus by consistent activation of pro-opiomelanocortins neurons increases melanocortin stimulating hormone (MSH) which is anorexigenic [29]. The overall increase in melanocortin increases anorexia and basal metabolism. In addition, cytokines affect the intestines instigating malabsorption. Pro-inflammatory cytokines impair the intestinal barrier by damaging the mucosa [30]. GDF-15 belonging to the TGF-beta family is involved in anorexia and weight loss [15].

Effect of cytokines on tumor growth and negative energy balance

A negative energy balance is when the energy requirement is significantly higher than the energy produced. Simply, the output does not meet the needs. This occurs because of an upsurge in the energy requirements of a growing tumor, metastasis, escalation of basal metabolic rate, the decline in energy provision to the body due to anorexia, and alteration in metabolism. TNF-α, IL-1-beta, IFN‐γ, and MCP-1-like cytokines acting via NF-kB are associated with a protein angiopoietin-like 4 (ANGPTL-4) which is produced majorly in adipose tissue as an adipokine and liver. This protein is involved in increasing the vascular supply to tumors by angiogenesis and increasing vascular permeability. By allowing the travel of tumor cells through endothelial cells, ANGPTL-4 also aids in tumor metastasis [16]. IL-6 is involved in increasing the tumor burden, and along with VEGF secreted by cancer cells, it is also involved in angiogenesis [31]. Every factor aiding in tumors ultimately causes an increase in energy expenditure.

Pro-inflammatory cytokines affect metabolism via numerous approaches. Changes caused by inflammatory cytokines in melanocortin change the way the body utilizes glucose and increase lipolysis [29]. WAT under systemic inflammation produces resistin. It affects glucose metabolism by increasing insulin resistance and also acts along with other inflammatory cytokines such as TNF-α, IL-6, and IL-10. Studies have found that an upsurge in the levels of resistin is associated with poor survival [32].

Effect of cytokines on the liver

IL-6 plays an important role in causing cancer cachexia. It changes protein production and synthesizes acute-phase proteins [9]. CRP levels are part of the evaluation of cancer cachexia. Inflammatory proteins and pro-inflammatory cytokines are continuous cycles stimulating each other. CRP induces white blood cell-like neutrophils and monocytes to release IL-6, TNF-α, and IL-6 [18]. These cytokines release thrombopoietin (THPO) from the liver [9].

Effect of cytokines on blood and bone marrow

Anemia is seen in one-third of cancer cachectic patients who are usually normocytic. Hepcidin is an acute-phase protein seen in response to systemic inflammation that decreases the absorption of iron in the gastrointestinal tract, thus decreasing the iron availability for the production of red blood cells. Pro-inflammatory cytokines also inhibit the production of erythropoietin from the kidney, thus reducing the production of erythroblasts from bone marrow. IL-6 along with other elements produces myeloid-derived suppressor cells directing to immune suppression. THPO produced from the liver produces platelets and is activated by IL-6 and IL-7 [9].

Future of cancer cachexia treatment

Although anti-cytokines are considered a potential future in the field of cancer cachexia, successful drugs are not available. This can be attributed to a lack of adherence due to disease progression, death, voluntary withdrawal, and drug toxicity. Thalidomide inhibits TNF-α, IL-6 and NF-kB have shown mixed results in drug trials; however, when given in combination with appetite stimulants megestrol acetate (MA) and medroxyprogesterone acetate (MPA) the results were better than those with only MA or MPA [33-37]. Certain anti-cytokines are in drug trials such as MAbetaP 1 which is an immunoglobulin G developed against IL-1, clazakizumab against IL-6, and 7E which is an anti-monoclonal antibody against IL-20 are under development for the treatment of cancer cachexia targeting various cytokines involved in its pathology [33,34,38]. Another orexigenic mediator Ghrelin along with increasing growth hormone-releasing hormone and insulin-like growth factor-1 inhibits cytokines IL-1-beta, IL-6, and TNF-α. It is also found to be inhibiting the NF-kB pathway and reducing MURF1 and MAFbx levels [35].

It is important to observe that even though single anti-cytokines have not been proven to be effective, drugs which inhibit multiple cytokines such as thalidomide and ghrelin containing anti-cancer or appetite stimulants are more effective in treating cancer cachexia. This is consistent with the observation that cancer cachexia is caused by a cascade of events involving multiple cytokines. Hence, it would be better to conduct more drug trials for combinations of anti-cytokines and anti-cytokines along with other drugs used in cachexia treatment.

Limitations

This is a literature review of only English-language papers. Though this review includes various ways cytokines affect the body in cancer cachexia, it does not discuss molecular mechanisms by which they bring these changes and the pharmacological outcomes of drugs acting against these cytokines. In addition, this review only included free and full-text papers published after 2002 into consideration.

## Conclusions

Cancer cachexia reduces the lifetime of cancer patients and decreases the quality of life. In addition, they have been proven to decrease the body’s response to chemotherapy in battling cancer. This study aims to understand the pathological role of pro-inflammatory cytokines in cancer cachexia. Currently, the majority of treatment of cachexia is about palliation. Drugs against inflammatory cytokines are being developed and are in different stages of trials for application in cachexia. With a clear picture of the role of multiple inflammatory cytokines in causing cancer cachexia, we hope drugs that can act on multiple inflammatory cytokine targets and combinations of anti-cytokines with other drugs can be developed and open a new avenue in the care and treatment of cancer patients.
